# Lung metastases formed by disseminated tumor cells exhibit different proliferation states: Micro-metastases with different growth rates

**DOI:** 10.3724/abbs.2024118

**Published:** 2024-07-08

**Authors:** Jiajun Liu, Shihui Liu, Jianhui Tian, Jiaxuan Li, Minghua Li, Zujun Que

**Affiliations:** 1 Institute of Oncology Shanghai Municipal Hospital of Traditional Chinese Medicine Shanghai University of Traditional Chinese Medicine Shanghai 200071 China; 2 Clinical Oncology Center Shanghai Municipal Hospital of Traditional Chinese Medicine Shanghai University of Traditional Chinese Medicine Shanghai 200071 China

Lung cancer is the leading cause of cancer mortality in China and worldwide [ 1, 2], and metastasis is the main cause of patient death [3]. Cancer cells invade and migrate from the primary tumor, enter the circulation system through intravasation, and become circulating tumor cells (CTCs). CTCs that survive in blood vessels extravasate and invade target organs to become disseminated tumor cells (DTCs). DTCs proliferate in target organs to metastasize to distant organs [3]. The previous view was that metastasis is the final stage of cancer progression. Normal cells first transform into tumor cells and then into invasive cancer cells; thus, metastasis occurs. Therefore, the possibility of metastasis is closely related to the size of the primary tumor. This is reflected in the TNM stage (T, tumor size; N, extent of spread to regional lymph nodes; M, metastasis to distant organs), which is often referenced in clinical diagnosis. However, an increasing number of studies are currently challenging this view. A previous study showed that the metastasis of malignant tumors occurs in the early stages of cancer. When patients are diagnosed with primary cancer, dissemination occurs. CTCs already exist in the blood vessels of early-stage lung cancer patients, and in early-stage lung cancer patients, DTCs are likely to be the main source of late-stage metastasis in some cancers [4]; they do not proliferate in target organs, so they cannot be eliminated by surgery, radiotherapy or chemotherapy. As a result, even if the lesions are removed through surgery in these patients, metastasis is still found months or years later, which affects the patient’s quality of life and reduces the patient’s survival period. These observations prompt scientists in the field of metastasis to pay more attention to the prevention and treatment of DTCs when formulating metastasis prevention strategies.

To determine whether DTCs exist in different states after entering the target organ, we used a mouse lung cancer metastasis model to generate CTC-TJH-01 cells [5], which are circulating tumor cells derived from the peripheral blood of early-stage lung adenocarcinoma patients who extravasate into target organs and become DTCs. Then, we observed the distribution and proliferation of DTCs in the lungs. Combined with traditional Chinese medicine theory, our findings can improve clinical medication regimens and promote innovations in metastasis prevention and treatment strategies.

**Figure 1:**
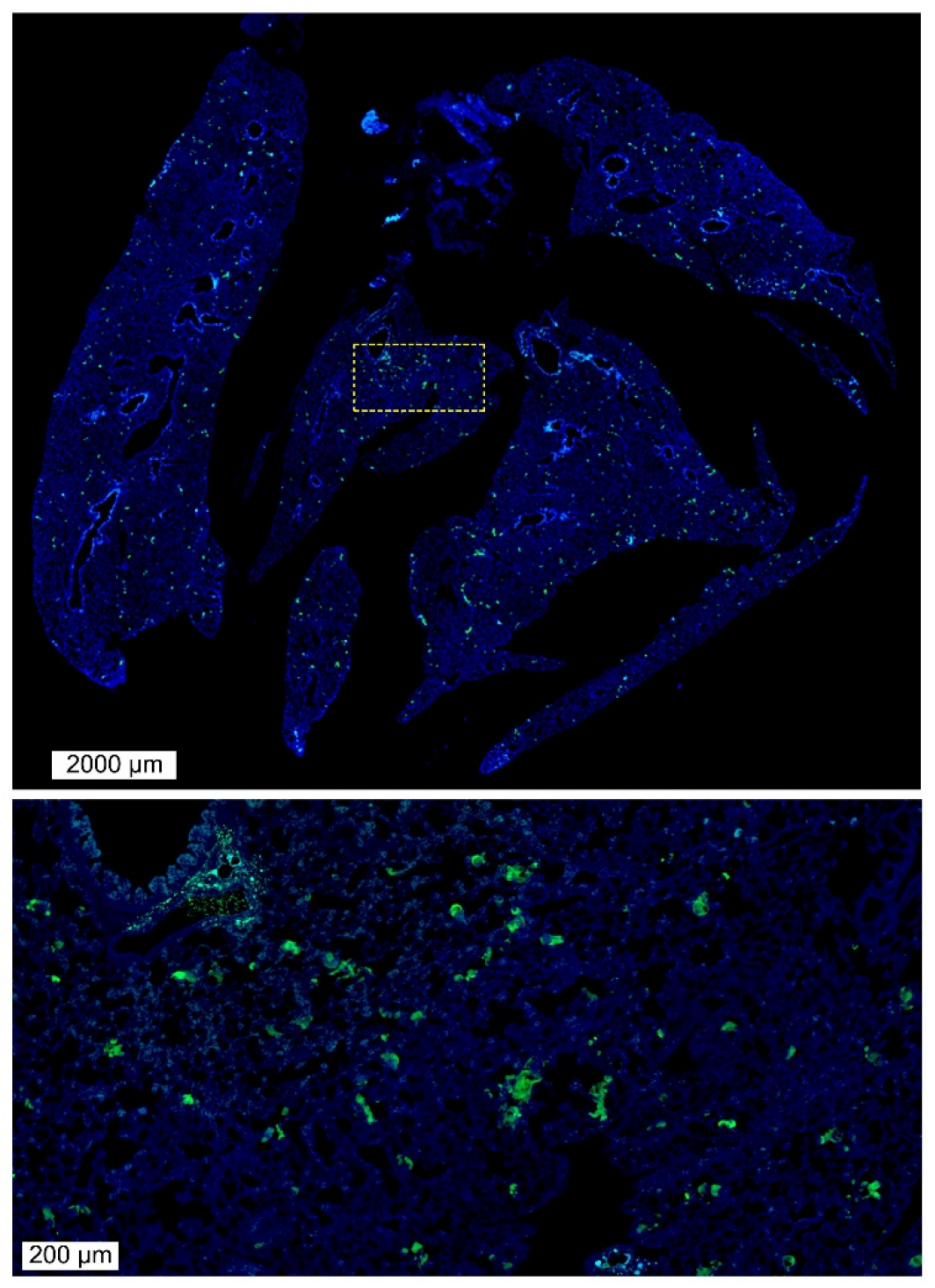
Figure 1 The spread of DTCs in the lungs of NOD/SCID mice 24 h after inoculation with CTCs A total of 5 × 105 CTC-TJH-01 cells were injected into the lateral tail vein of NOD/SCID mice, and vimentin was used as a lung tumor marker. Immunofluorescence staining was performed, and CTC-TJH-01 cells that reached the lungs through the peripheral circulation were evenly spread over 24 h (1× and 10× magnification of fluorescence microscopy).

We observed the potential distribution and proliferation status of DTCs in the lungs in a mouse lung cancer metastasis model. A lung colonization assay was performed by injecting 5 × 10 ^5^ CTC-TJH-01 cells into the lateral tail vein of NOD/SCID mice, and vimentin was used as a lung tumor marker. Immunofluorescence staining was performed, and CTC-TJH-01 cells that reached the lungs through the peripheral circulation were evenly spread over 24 h. This finding showed that cancer cells can move to distant sites through the circulation, especially the lungs, which are rich in blood vessels, and stay there in the form of DTCs ( Figure 1).

**Figure 2:**
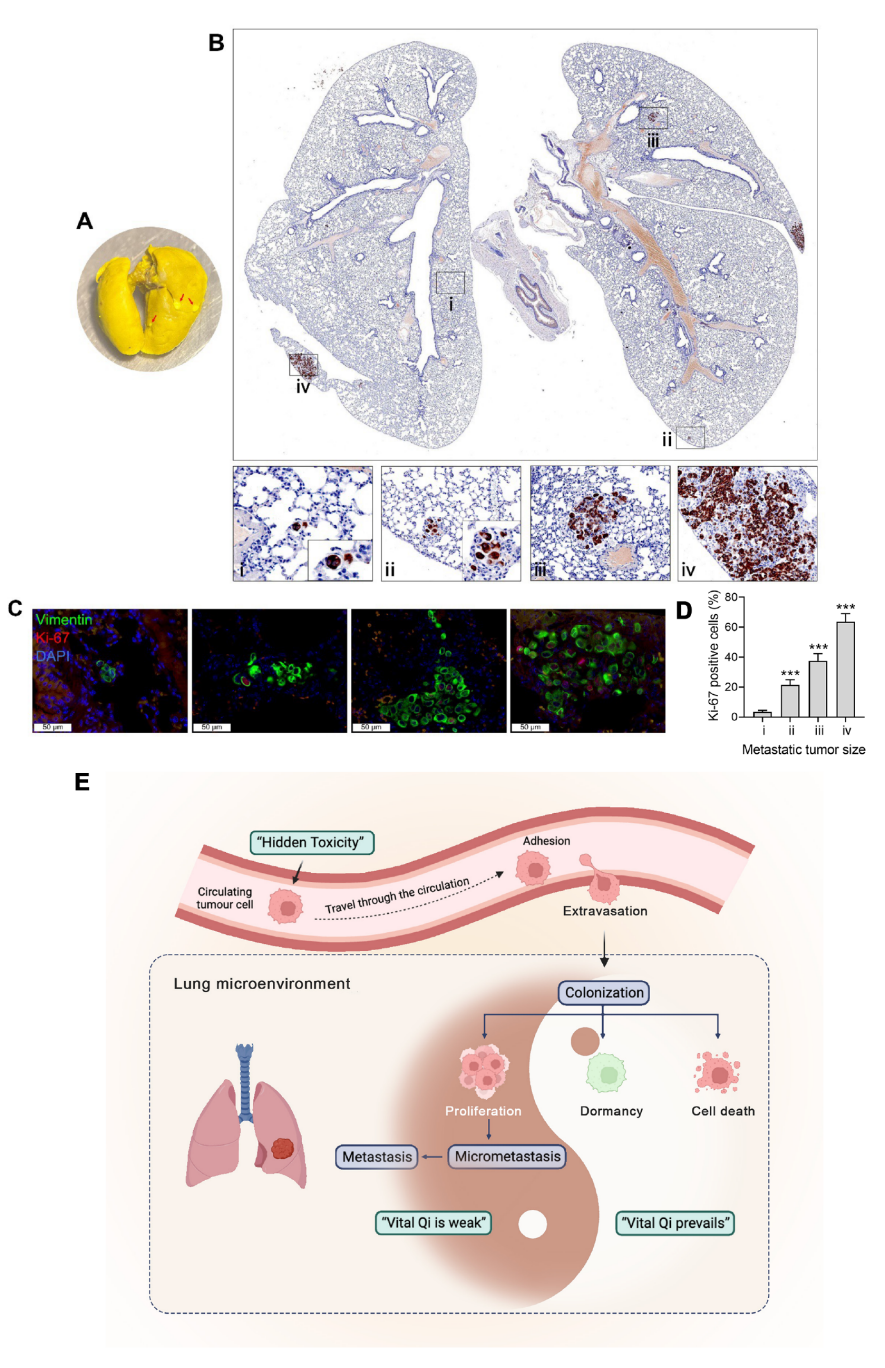
Figure 2 Distribution and proliferation of DTCs in the lungs of NOD/SCID mice 12 weeks after inoculation with CTCs (A) Lungs of NOD/SCID mice. (B) Immunohistochemistry was used to detect vimentin to indicate tumor cells in mouse lungs. (C) Immunofluorescence staining of Vimentin and Ki67 was used to observe the proliferation of metastatic cells in the lungs. Scale bar: 50 μm. (D) Quantification of Ki67 immunofluorescence. (E) Schematic diagram of the “hidden toxicity due to vital Qi deficiency” theory, which summarizes this phenomenon. The data are shown as the mean ± SD. n = 3. ***P < 0.001 vs the i group. i, ii, iii, and iv represent different sizes of metastatic lesions.

However, after 12 weeks, there were only a few visible metastases in the lungs ( Figure 2A), and many tumor cells in the visible metastases were Ki67-positive ( Figure 2C). Immunohistochemistry revealed other Vimentin-positive tumor cells in the lungs ( Figure 2B), but as the number of cells decreased, the Ki67 positivity rate also decreased, and a single tumor cell was negative for Ki67 ( Figure 2C,D). This finding shows that an unsuitable microenvironment induces DTC apoptosis, and only a very small number of DTCs mediate the formation of an immunosuppressive microenvironment and then proliferate to form metastatic lesions. In addition, DTCs that survive have different proliferation rates; some proliferate to form metastases, while others remain dormant somewhere as individuals. These metastases of different sizes that coexist in the lungs may also have different responses to radiotherapy and chemotherapy due to their different proliferation rates. This may also explain why early-stage lung cancer patients still develop metastasis after standard clinical treatment.

Before disseminated tumor cells proliferate and form visible metastases, they generally cannot be detected clinically through conventional diagnostic methods or tumor biomarkers, and patients at this stage often have no clinically significant symptoms; this stage can be called the “metastasis subclinical stage”. Tian *et al*. [6] proposed the pathogenesis theory of “hidden toxicity due to vital Qi deficiency” for this stage of lung cancer metastasis. According to this theory, DTCs in a dormant state are already present in the metastatic target organs of patients with early-stage lung cancer after surgery. Immunosenescence or stress mediates immune dysfunction, leading to the activation and proliferation of dormant DTCs, which in turn leads to the occurrence of clinical metastasis. Our study revealed different states of “hidden toxicity” in the target organs of metastasis at the same time ( Figure 2E).

In previous studies, we revealed the expression patterns of myeloid-derived suppressor cells, regulatory T cells, and circulating tumor cells in lung cancer patients at this stage and established the human lung adenocarcinoma CTC cell line CTC-TJH-01 [5]. We found that in the process of cancer metastasis, there is a struggle between “vital Qi” and “hidden toxicity”. If vital Qi prevails, hidden toxicity will be subdued without causing disease; however, if vital Qi is weak, the efficiency of immune surveillance and immune clearance decreases, and hidden toxicity will burst out to cause disease. Therefore, vital Qi in the body is the key to determining whether metastasis will eventually occur. Comprehensively regulating vital Qi from the perspective of the body, energy, and spirit has become an important strategy for the prevention and treatment of cancer metastasis.

Under the guidance of this theory, we found that TCM-derived polyphyllin VII plays an important role in targeting “hidden toxicity” CTCs [7]. We also found that the traditional Chinese medicine compound Jinfukang, which is commonly used in the clinical treatment of lung cancer, can affect the proliferative state of “hidden toxicity” CTCs directly or by regulating immune cells [ 8, 9]. In clinical practice, many patients can achieve long-term survival with tumors with the help of TCM, which suggests that the anti-metastasis effect of TCM is likely to be based on maintaining “hidden toxicity” in metastatic target organs in a long-term dormant state. In this study, we found that when metastasis occurs in the early stage, a large number of CTCs may be distributed in target organs ( Figure 1), but most of these cells die due to apoptosis or anoikis. Very few CTCs remained in target organs after extravasation and successfully survived to become DTCs ( Figure 2B). Among these DTCs, a single DTC was negative for Ki67 expression and was in a dormant state. As the number of DTCs increased, there were more Ki67-positive cells ( Figure 2C,D). These findings confirmed that more DTCs were present in the target organs before macroscopic metastasis occurred. The occurrence of metastasis is the result of the transition of dormant DTCs from dormancy to proliferation under the influence of immune disorders in the tumor microenvironment. As Barkley *et al*. [10] showed in their research, cancer cells can form specific interactions with the tumor microenvironment. Once the immunosuppressive microenvironment mediated by tumor cells is formed, these tumor cells are out of control and are activated from the dormant state to the proliferative state and then proliferate to form metastatic lesions. At the same time, due to differences in the microenvironments at different locations, the awakening time of tumor cells varies, which leads to the formation of four states, I‒IV, in the same metastatic organ. The differences in the microenvironments of different lesions and the differences in the growth rates of tumors may indicate differences in drug sensitivity to therapeutic drugs. Based on these findings, we speculate that maintaining DTCs in a dormant state, rather than by inhibiting the invasion and migration of primary tumor cells, may be an effective strategy for preventing lung cancer metastasis.

In conclusion, this study simulated the metastasis process of lung cancer cells through a mouse model, visualized the different stages of DTCs during tumor metastasis, and indicated the proliferation rates of DTCs at different stages, thus proving that metastasis does not need to follow a strict time sequence. These results support the traditional Chinese medicine theory of “hidden toxicity due to vital Qi deficiency” previously proposed by us. This theory fits the concept of the tumor microenvironment and the rise of cancer immunotherapy, provides a new perspective on traditional Chinese medicine in cancer treatment, and provides a reference for solving the clinical problem of metastasis.
